# Electroacupuncture-Induced Muscular Inflammatory Pain Relief Was Associated With Activation of Low-Threshold Mechanoreceptor Neurons and Inhibition of Wide Dynamic Range Neurons in Spinal Dorsal Horn

**DOI:** 10.3389/fnins.2021.687173

**Published:** 2021-07-08

**Authors:** Cheng-Lin Duan-Mu, Xiao-Ning Zhang, Hong Shi, Yang-Shuai Su, Hong-Ye Wan, Yi Wang, Zheng-Yang Qu, Wei He, Xiao-Yu Wang, Xiang-Hong Jing

**Affiliations:** Institute of Acupuncture and Moxibustion, China Academy of Chinese Medical Sciences, Beijing, China

**Keywords:** electroacupuncture, muscular inflammatory pain, complete Freund’s adjuvant, low threshold mechanoreceptor neuron, wide dynamic range neuron

## Abstract

Acupuncture is an effective alternative therapy for pain management. Evidence suggests that acupuncture relieves pain by exciting somatic afferent nerve fibers. However, the mechanism underlying the interaction between neurons in different layers of the spinal dorsal horn induced by electroacupuncture (EA) remains unclear. The aim of this study was to explore the mechanism of EA relieving inflammatory muscle pain, which was associated with activation of the spontaneous firing of low-threshold mechanoreceptor (LTM) neurons and inhibition of wide dynamic range (WDR) neuronal activities in the spinal dorsal horn of rats. Inflammatory muscle pain was induced by injecting complete Freund’s adjuvant into the right biceps femoris muscle. EA with intensity of threshold of A fibers (Ta) in Liangqiu (ST34) muscle considerably inhibited the abnormal spontaneous activities of electromyography (EMG) due to muscle inflammation. While EA with intensity of C-fiber threshold (Tc) increased the abnormal activities of EMG. EA with Ta also ameliorated the imbalance of weight-bearing behavior. A microelectrode array with 750-μm depth covering 32 channels was used to record the neuronal activities of WDR and LTM in different layers of the spinal dorsal horn. The spontaneous firing of LTM neurons was enhanced by EA-Ta, while the spontaneous firing of WDR neurons was inhibited. Moreover, EA-Ta led to a significant inverse correlation between changes in the frequency of WDR and LTM neurons (*r* = −0.64, *p* < 0.05). In conclusion, the results indicated that EA could alleviate inflammatory muscle pain, which was associated with facilitation of the spontaneous firing of LTM neurons and inhibition of WDR neuronal activities. This provides a promising evidence that EA-Ta could be applied to relieve muscular inflammatory pain in clinical practice.

## Introduction

Acupuncture has been widely applied as an effective intervention for pain relief in numerous clinical trials and experimental studies over the last several decades (Hauck et al., 2017; Kwon et al., 2017; Yu et al., 2020). As a mechanical stimulation applied to the body surface, acupuncture activates somatic afferents to generate action potentials that send signals from the peripheral to the central nervous system (CNS) (Kagitani et al., 2010). In fact, neurons in multiple spinal and brain regions associated with pain modulation are responsive to acupuncture stimulation (Zhao, 2008; Guo et al., 2020). It is believed that the analgesic effect of acupuncture, at least partially, results from the integration between nociceptive inputs and acupuncture-induced impulses at different CNS levels.

The spinal dorsal horn serves as the first relay station in the CNS for the transmission, integration, and modulation of both innocuous and nociceptive signals (Wang et al., 2012; Peirs et al., 2015; Cordero-Erausquin et al., 2016; Zhou et al., 2019). Somatosensory inputs are conveyed *via* primary afferents to the superficial and deep dorsal horn neurons, forming mono- or polysynaptic connections (Woolf and Fitzgerald, 1986; Todd, 2010; Punnakkal et al., 2014). Based on the sensitivity to mechanical stimulation, dorsal horn neurons are classified as low-threshold mechanoreceptive (LTM), nociceptive-specific (NS), and wide-dynamic range (WDR) neurons, which play different roles in the processing of distinct sensory modalities. Accumulating evidence indicated that WDR neurons are an integral component of the ascending nociceptive pathway and appear to be sensitized in models of neuropathic, inflammatory, and osteoarthritic pain (McGaraughty et al., 2018). In complete Freund’s adjuvant (CFA)-treated rats, WDR neurons tended to display greater spontaneous firing and enhanced excitability, which accounted for the mechanical hypersensitivity of the inflamed muscle (McGaraughty et al., 2006; Kiyomoto et al., 2015). Notably, in addition to peripheral input, WDR neurons also receive excitatory or inhibitory input from the intraspinal level (Zain and Bonin, 2019), which provides a neuronal basis for pain modulation in local circuits. LTM neurons mainly receive sensory inputs from A fibers to encode innocuous mechanical signals and may participate in pain modulation as well. A previous study of craniofacial pain revealed that cannabinoid receptor agonist-induced analgesia involved both the inhibition of WDR neurons and the facilitation of LTM neurons (Papanastassiou et al., 2004). Additionally, LTM neurons showed prolonged excitation, whereas WDR neurons exhibited prolonged inhibition following the application of the potent analgesic clonidine (Millar et al., 1993). However, it is still inconclusive whether these analgesic effects are attributed to LTM neuron-induced inhibition of WDR neurons.

The gate control theory of pain proposed by Melzack and Wall (1965) argues that low-threshold Aβ-inputs suppress spinal nociceptive transmission *via* feed-forward inhibition. Consistently, selective stimulation of primary afferent A-fibers inhibited the C-fiber-evoked activity of deep laminae WDR neurons in the spinal dorsal horn (Mendell, 1966; Woolf and Wall, 1982). Acupuncture analgesia is thought to be partially achieved by A-fiber-mediated “gate” control of noxious inputs (Zhao, 2008). It has been reported that electroacupuncture (EA) could modulate somatic afferents to inhibit the noxious WDR neuronal activity in migraine rats (Qu et al., 2020). Therefore, these findings provide a possibility that EA with low intensity may attenuate WDR neuron-mediated pain transmission by activating LTM neurons. In the present study, by measuring the weight-bearing and EMG activity of the inflamed muscle, we verified the analgesic effect of EA with different intensities in CFA-treated rats firstly. Subsequently, the responses of LTM and WDR neurons to EA stimulation were recorded using a microelectrode array in the spinal dorsal horn. We further performed coherence analysis to determine the interplay between LTM and WDR neurons corresponding to EA stimulation, so as to elucidate a potential spinal neuronal substrate for EA analgesia in CFA-induced inflammatory conditions.

## Materials and Methods

### Animals

Male Sprague–Dawley (SD) rats, weighing 200–250 g, were purchased from the Institute of Laboratory Animal Science, Chinese Academy of Medical Sciences [experimental animal license number: SCXK (Jing) 2017-0013]. The experimental procedures were approved by the Ethics Committee of the Institutional Animal Welfare and Use Committee of Acupuncture and Moxibustion Institute of China Academy of Chinese Medical Sciences (No. D2018-04-13-1). All manipulations were performed in accordance with the recommendations of the Guide for Care and Use of Laboratory Animals issued by the National Institutes of Health. Efforts were made to minimize the number and suffering of the animals used. The animals were maintained under a standard 12-h light–dark cycle with free access to food and water and allowed to acclimate to the housing conditions for 7 days prior to the experiment.

### Complete Freund’s Adjuvant Injection

Under anesthesia with 2% isoflurane (0.5 L/min, RWD Life Science, China), the right biceps femoris was injected with 200 μl of CFA (F5881, Sigma, United States) composed of inactivated and dried mycobacteria. CFA was injected slowly into the muscle with no leakage observed. The left (control) biceps femoris received 200 μl of sterile saline.

### Measurement of Weight-Bearing

To assess the severity of inflammatory pain, we measured the differences in weight-bearing capacity between the right (experimental) and left (control) hindlimb using a non-invasive pain bipedal balance instrument (IITC Incapacitance Analgesia Meter, IITC Life Science Inc., Canada). Each rat was placed in an individual plexiglass with each hind paw resting on a separate force plate. The weight-bearing capacity (g) of each hindlimb in 10 s was automatically averaged. Each data point was considered as the difference score (g) over the left (control) minus right (experimental) hindlimb. The tests were repeated three times and the mean values were calculated.

### Recording of Electromyographic Activity

Electromyographic (EMG) activities were recorded on the fourth day after CFA injection *via* a pair of needle electrodes inserted into the biceps femoris muscle under anesthesia with isoflurane. EMG signals were captured and amplified online by a bioelectric amplifier (DigitimerNL900D, Digitimer, United Kingdom) and a data acquisition system (PowerlabPL3508, AD Instruments, Australia). After recording stable EMG activity for 5 min, acupuncture stimulation was applied to ST34 and EMG activity was monitored for an additional 5 min. The area under the curve (AUC) and the inter-spike intervals (ISI) of spontaneous EMG activities were calculated through the Spike 2 software (version 7, CED, United Kingdom). EMG activity was determined from the mean AUC and ISI within 1 min before and after EA.

### Electroacupuncture Stimulation

Under anesthesia with 2% isoflurane, rats were treated with EA on the fourth day after CFA injection. Liangqiu (ST34) was selected in this study, because it has been widely used in pain relief (Shen et al., 2015). ST34 is located at 5 mm superior and lateral to the knee joint. EA was delivered by an electrical stimulator (PowerlabFE180, AD Instruments, Australia) *via* a pair of stainless needles (0.18 mm diameter, 13 mm length; Beijing Zhongyan Taihe Medicine Co., China) inserted into ipsilateral ST34 (square waves; frequency: 2/100 Hz) for 1 min *in vivo* electrophysiological recording. For studying the effect on the imbalance of weight-bearing in CFA rats, EA was performed for 30 min.

The intensities of EA used in this study were identified as the thresholds of the A- and C-fiber activation. The ipsilateral biceps femoris muscle reflex was evoked by electrical stimulation of the A- and C-fibers within the receptive field of the sural nerve (Guirimand et al., 1994; Zhi et al., 2017). Specifically, EMG responses were recorded *via* a pair of needles inserted into the biceps femoris muscle. Stimulating electrodes were inserted into the lateral part of the fourth and fifth toes within the field of sural nerve innervation. Six single-square waves of 1-ms duration from an electrical stimulator (PowerlabFE180, AD Instruments, Australia) were delivered at a frequency of 0.2 Hz. The threshold of A fibers (Ta) and C-fiber threshold (Tc) were considered as the minimal stimulation intensities required to evoke EMG of A- or C-fiber activities with the conductive velocity of 4–36 or 0.4–2 m/s, respectively (Duanmu et al., 2020).

### Recording of the Spinal Dorsal Horn Neurons

#### Surgery

**FIGURE 1:**
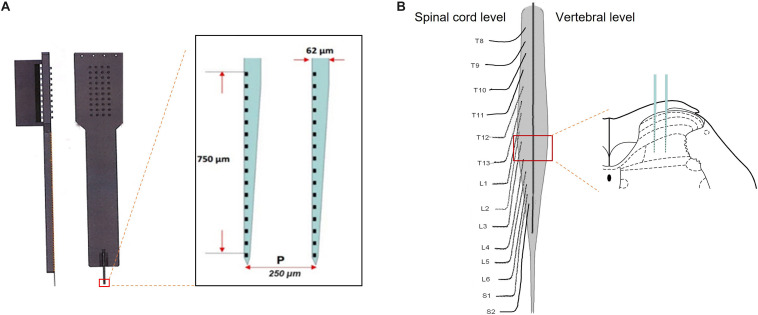
Microelectrode array was performed on the spinal lumbar enlargement to record firing of neurons across the spinal dorsal horn. Panel **(A)** illustrated a microelectrode array, consisting of two electrodes with 750-μm depth covering 32 channels. Following the removal of the dorsal aspect of the vertebrae and meninges, the microelectrode array was inserted vertically into the L3–4 level to record firing of the neurons in different layers of the spinal dorsal horn **(B)**.

After being anesthetized with 10% urethane (1.2 g/kg, i.p.), the lumbar enlargement of the spinal cord (L3–L4 level) was exposed by a laminectomy at the T12–L1 vertebrae, and the dura mater of lumbosacral spinal segments was carefully removed. The corresponding vertebral column was tightly fixed in a frame with clamps. All exposed spinal segments were covered with 37°C artificial cerebrospinal fluid (aCSF, CZ0516, Leagene, China) during surgery and recording. Core body temperature was monitored and maintained at 37.0 ± 0.5°C through a feedback-controlled electric blanket (ALC-HTP, Shanghai Alcott Biotech Co., China). A microelectrode array (ASSY, Lotus Biochips, United States) was inserted vertically into L3/4 to a depth of approximately 1,000 μm using a micromanipulator (DMA-1510, Narishige, Japan) (Figures 1A,B). The reference electrode was placed in the nearby muscle. The array was attached to the headstage using a custom connector. Signals were amplified by a preamplifier (LB-0164-1, Blackrock Microsystems, United States) with a bandwidth of 250 Hz–5 kHz. A microelectrode array was used to record neuronal activities, and the signals were captured and amplified online by a data acquisition system (Cerebus-128, Blackrock Microsystems, United States). An overdose of 20% urethane (3 g/kg, i.p.) was used to euthanize the rats following completion of the experiment.

#### Identification of Receptive Fields and Neuron Classification

The receptive fields (RFs) of the neurons were identified with a mechanical press stimulator (ALMEMO2450, Ahlborn, Germany). Muscular nociceptive neurons in CFA rats were evoked by 200-mN pressure stimulation when there was no response to 60-mN pressure stimulation, as described previously (Fang et al., 2020). Therefore, innocuous stimulation (60 mN) and noxious stimulation (200 mN) were chosen to identify WDR neurons and LTM neurons in the spinal dorsal horn, respectively. To ensure that the RFs were correctly located in the muscle (instead of the skin), we applied the same pressure stimulation to the skin. In neurons that responded to innocuous or noxious pressure stimulation applied to the muscle with no response to brushing and pinching of the covering skin, the RFs were considered to be in the muscle (Hoheisel et al., 2015). Neurons were defined as LTM or WDR based on their response to innocuous (60 mN) or noxious (200 mN) pressure stimulation applied to the receptive field for 10 s. LTM neurons were defined as cells that responded to innocuous stimulation instead of noxious stimulation. WDR neurons responded to both innocuous (60 mN) or noxious (200 mN) stimulation. After the neurons were identified, persistent recording was performed for at least 3 min without any external stimulation. If the spontaneous firing was ongoing, the neurons were defined as spontaneously active. The frequency of spontaneous firing and changes in the frequency in 1 min were analyzed. Changes in the average frequency were calculated according to the following equation: (the value after EA−the value before EA)/the value before EA × 100%. If the changes in the frequency of spontaneous activity after EA were more than 20% of the basal activity, the response was considered to have an excitatory or inhibitory effect.

### Statistical Analysis

All data are expressed as mean ± standard error of the mean. Statistical analysis was performed with the SPSS 23 software. The Shapiro–Wilk test was used to evaluate whether these groups fit the normal distributions. Normally distributed groups were analyzed *via* parametric tests. Differences among multiple groups were analyzed with one-way analysis of variance (ANOVA) test followed by the LSD, SNK, or Dunnett’s T3 *post hoc* test. Non-normally distributed groups were analyzed with non-parametrical tests. The data obtained before and after EA in the same group were compared statistically with Wilcoxon test. Correlation analysis was performed through Pearson Product-Moment Correlation. *p* < 0.05 was considered a statistical significance.

## Results

### Spontaneous EMG Activity and Imbalance of Weight-Bearing Induced by Injection of CFA Into Biceps Femoris

**FIGURE 2:**
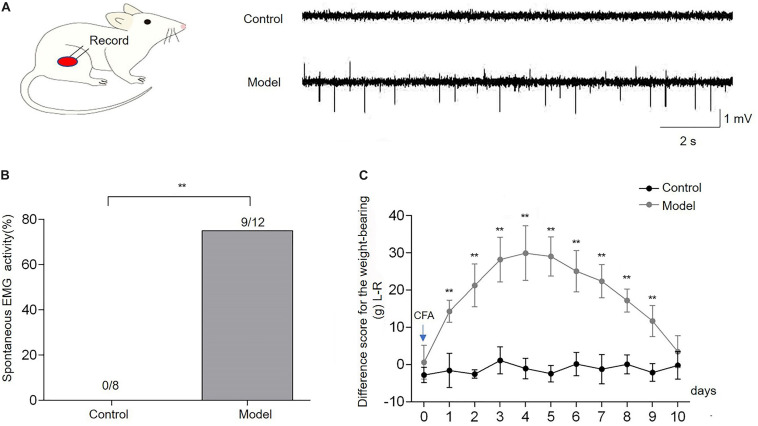
Complete Freund’s adjuvant (CFA) injection induced the imbalance of weight-bearing and spontaneous electromyography (EMG) activities of the biceps femoris. Spontaneous EMG activities of the biceps femoris were observed in the model group, while there was no spontaneous EMG activity in control rats **(A,B)**. The difference score for the weight-bearing (L−R) markedly increased from the first day after CFA injection into the right biceps femoris muscle and persisted for 9 days in comparison with the baseline (day 0) **(C)**. Data are expressed as mean ± SEM (**p < 0.01).

After injection of CFA into the right biceps femoris, severe edema of the ipsilateral hindlimb was observed. The spontaneous EMG activity of the biceps femoris was recorded to confirm the development of muscle pain in the CFA-inflamed hindlimb. It was shown that spontaneous EMG activity occurred in 9/12 of CFA-treated rats during 4–5 days after CFA, whereas no spontaneous EMG activity was observed in control rats (Figure 2A). The incidence of spontaneous EMG activity in CFA-injected rats (*n* = 12) was significantly higher compared with the control rats (*n* = 8, *p* < 0.01, Figure 2B). The weight-bearing was evaluated in unrestrained rats to assess the pain-related guarding behavior. Compared with the control group, the difference score for the weight-bearing (L - R) was increased markedly from the first day after CFA injection (*p* < 0.01, *n* = 10, Figure 2C), and it persisted for 9 days (*p* < 0.01), indicating that CFA-treated rats suffered persistent muscle pain and placed significantly less weight on the ipsilateral CFA-inflamed hindlimb. These data demonstrated that CFA produced reliable inflammatory muscle pain in the affected hindlimb, as indicated by the increased difference score for the weight-bearing and spontaneous EMG activities, which was consistent with previous studies (Duan et al., 2013; Fang et al., 2020).

### Quantification of the Intensities of EA-Ta and EA-Tc by a C-Fiber Reflex

**FIGURE 3:**
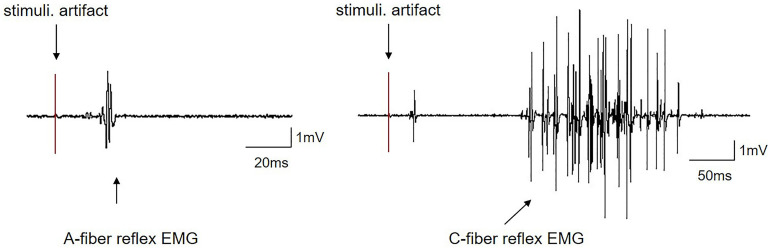
Representative traces of EMG induced by A-fiber or C-fiber reflex in CFA rats.

The intensities of EA stimuli for activating A- or C-fibers were determined according to their corresponding thresholds (Ta or Tc) measured by the evoked A- and C-fiber reflexes as described previously (Falinower et al., 1994; Zhu et al., 2004). The typical reflex of EMG responses elicited by A- and C-fibers is shown in Figure 3, which could be apparently distinguished according to their electrophysiological properties. Generally, A-fiber reflex possessed a shorter latency (24.7 ± 1.74 ms), smaller duration (9.84 ± 1.61 ms), and lower threshold (1.27 ± 0.11 mA), while C-fiber reflex had a relatively longer latency (174.02 ± 8.22 ms), greater duration (199.84 ± 10.86 ms), and higher threshold (3.81 ± 0.27 mA) (Table 1). The conductive velocity of A-fiber and C-fiber in CFA rats was calculated from the distances between the stimulus and recording sites dividing stable latency, respectively (Ta: 6.92 ± 0.71 m/s; Tc: 0.95 ± 0.04 m/s). Based on these characteristics, we defined the current intensities of EA-Ta and EA-Tc as 1.3 and 4 mA, respectively, in the present study.

**TABLE 1:** Threshold, latency, and duration of Aδ- and C-fiber reflex in complete Freund’s adjuvant rats (*n* = 12).

	**Threshold (mA)**	**Latency (ms)**	**Duration (ms)**
A-fiber reflex	1.27 ± 0.11	24.7 ± 1.74	9.84 ± 1.61
C-fiber reflex	3.81 ± 0.27	174.02 ± 8.22	199.84 ± 10.86

A-fiber and C-fiber stimulation induced independent EMG responses. The early (A-fiber reflex) component and late (C-fiber reflex) component responses exhibited a difference in latency. The first component was a single di- or triphasic response with a short duration and low threshold. The second component was generally multiphasic with a longer duration and a higher threshold. Stimuli. A- or C-fiber threshold intensity stimulation.

### EA-Ta Inhibited the Spontaneous EMG Activities and Alleviated the Imbalance of Weight-Bearing in CFA Rats

**FIGURE 4:**
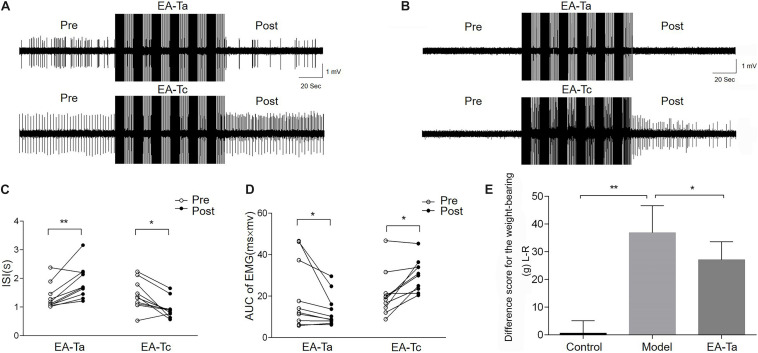
Electroacupuncture-threshold of A fibers (EA-Ta) applied to ST34 inhibited the spontaneous EMG activities while EA-Tc deteriorated the abnormal EMG activities in CFA rats. Panels **(A,B)** were the representative traces of spontaneous EMG activities before and after EA-Ta and EA-Tc in CFA and control rats. The area under the curve (AUC) and inter-spike intervals (ISI) of spontaneous EMG activities were elicited obviously before EA in CFA rats. EA-Ta applied to ST34 considerably inhibited the AUC and increased the ISI of spontaneous EMG activities in CFA rats, while EA-Tc deteriorated the EMG activities in CFA rats **(A)**. No spontaneous EMG activity was observed in control rats. The AUC and ISI showed no obvious change after application of EA-Ta. However, EA-Tc elicited the abnormal EMG activities in control rats **(B)**. The AUC was significantly inhibited and ISI were increased in EMG activities after EA-Ta, while EA-Tc noticeably aggravated the abnormal EMG in CFA rats **(C,D)**. EA-Ta obviously ameliorated the imbalance of weight-bearing compared with the CFA rats **(E)**. *n* = 10 in each group. Data are expressed as mean ± SEM (^∗∗^*p* < 0.01; *p < 0.05).

On the fourth day after CFA injection, EA-Ta and EA-Tc at ST34 were applied separately to verify the analgesic effect of EA on CFA-induced inflammatory pain. The AUC and ISI of spontaneous EMG activity were measured before and after EA. Figures 4A,B shows representative traces of spontaneous EMG activities altered by EA-Ta or EA-Tc stimulation in CFA and control rats. EA-Ta applied to ST34 considerably inhibited the spontaneous EMG activities in CFA rats, and the EMG activities showed no obvious change after application of EA-Ta in control rats. However, EA-Tc deteriorated the EMG activities in the control and CFA rats. We found that EA-Ta significantly reduced the AUC and increased the ISI of EMG in CFA rats compared with the baseline (Pre-EA) (post vs. pre: 13.26 ± 2.53 vs. 20.53 ± 5.17 mv.ms, *p* < 0.05; 1.87 ± 0.18 vs. 1.35 ± 0.14 s, *p* < 0.01, Figures 4C,D). However, the spontaneous EMG activity was noticeably exacerbated following EA-Tc (post vs. pre: 30.06 ± 2.45 vs. 20.94 ± 3.45 mv.ms, *p* < 0.05; 0.95 ± 0.11 vs. 1.42 ± 0.16 s, *p* < 0.05, Figures 4C,D). Moreover, the difference score for the weight-bearing in model rats was significantly higher than that of the control (model group vs. control group: 36.89 ± 9.71 vs. 0.62 ± 4.46 g, *p* < 0.01), but EA-Ta markedly attenuated the imbalance compared with the model group (EA-Ta vs. model group: 27.09 ± 6.52 vs. 36.89 ± 9.71 g, *p* < 0.05, Figure 4E). Therefore, these results suggested that EA with lower intensity to activate A-fibers was effective in relieving CFA-induced inflammatory pain, but stronger EA stimulation was likely to exaggerate local pain signaling when activating C-fibers. Therefore, EA-Ta was an appropriate intensity to relieve inflammatory muscle pain.

### EA-Ta Enhanced the Activity of LTM Neurons and Inhibited WDR Neurons in CFA Rats

**FIGURE 5:**
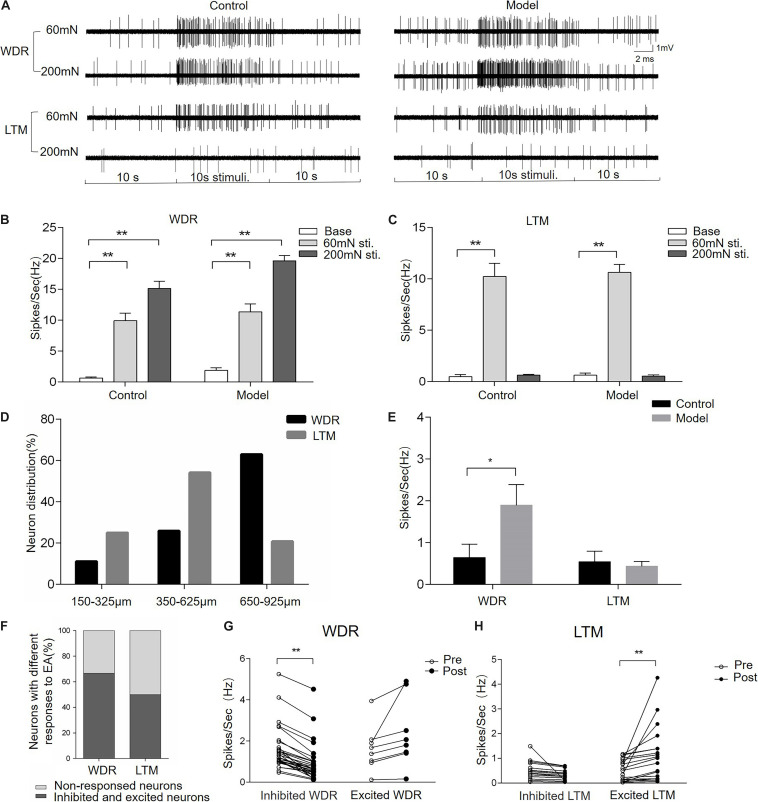
Electroacupuncture-threshold of A fibers inhibited the spontaneous firing of wide dynamic range (WDR) neurons and facilitated low-threshold mechanoreceptor (LTM) neurons. Panel **(A)** displayed the typical traces of LTM and WDR neurons responsible for mechanical stimulation in control and CFA rats. The spontaneous firing of WDR neurons was enhanced by 60- and 200-mN pressure stimulations, while the LTM neuron only responded to 60 mN. The frequency of the evoked firing of WDR and LTM neurons was significantly increased in the control and model group compared with the baseline **(B,C)**. The percentage of WDR and LTM neurons at different depths of the spinal dorsal horn is shown in panel **(D)**. Most of the LTM neurons were located at a depth of 350–625 mm and WDR neurons were mainly distributed at a depth of 650–925 mm from the surface of the spinal cord **(D)**. The frequency of spontaneous firing of WDR neurons significantly increased in the model group compared with the control. However, there was spontaneous firing of LTM neurons in either CFA or control rats **(E)**. EA-Ta produced the spontaneous firing of LTM and WDR neurons enhanced, inhibited, or non-response; 66.7% of WDR neurons and 50% of LTM were excited or inhibited, while others showed non-response to EA-Ta **(F)**. The frequency of spontaneous firing of WDR neurons was enhanced, while LTM neurons were significantly facilitated by EA-Ta **(G,H)**. Data are expressed as mean ± SEM (**p < 0.01; *p < 0.05).

To uncover the neuronal substrate of EA-Ta-induced analgesia, microelectrode array recording was performed on the spinal lumbar enlargement to determine the responses of dorsal horn neurons to EA-Ta in CFA-treated rats. We firstly identified neurons in different layers of the spinal dorsal horn with a mechanical press stimulator. WDR neurons were characterized as responding to both innocuous 60-mN and noxious 200-mN mechanical stimulation of the biceps femoris, while LTM neurons only responded to 60-mN stimulus (Figure 5A). The frequency of evoked firing of WDR neurons was significantly higher during response to 60- and 200-mN mechanical stimulation of the biceps femoris compared with the baseline in control and CFA-treated rats, and the evoked firing of LTM neurons was enhanced by 60-mN pressure stimulations (*p* < 0.01) (Figures 5B,C). A total of 92 neurons were recorded from eight control rats and eight model rats, among which 56 were identified as WDR neurons and 36 were LTM neurons. Figure 5A displayed representative firing traces of LTM and WDR neurons elicited by corresponding stimulation of the biceps femoris (RFs) in control and CFA-treated rats. LTM neurons were predominantly located at a depth of 350–625 mm (laminae III–IV) and WDR neurons were mainly distributed at a depth of 650–925 mm (laminae V) from the surface of the spinal cord (Figure 5D). These distribution patterns were in agreement with previous findings (McGaraughty et al., 2010; Xu et al., 2012; Schuelert et al., 2015). We found that, compared with control, the frequency of spontaneous firing of WDR neurons significantly increased in CFA rats (model group vs. control group: 1.89 ± 0.49 vs. 0.64 ± 0.31 Hz, *p* < 0.05), whereas LTM neurons showed no obvious change in firing frequency (Figure 5E). The alteration of spontaneous firing of LTM and WDR neurons were recorded before and after EA-Ta. Neurons with more than 20% changes in the frequency of spontaneous firing were considered as the responsive neurons, which showed excitatory or inhibitory response to EA-Ta stimulation. A total of 57 WDR and 72 LTM neurons were recorded in the 30 CFA-treated rats. It turned out that approximately 66.7% (38/57) of WDR neurons were identified as responsive neurons to EA-Ta stimulation, in which 78.9% (30/38) of responsive WDR neurons were significantly suppressed (post vs. pre: 0.90 ± 0.17 vs. 1.60 ± 0.19 Hz, *p* < 0.01). Only 21.1% (8/38) of responsive WDR neurons were slightly excited following EA-Ta stimulation, but no significant difference was observed (post vs. pre: 2.38 ± 0.59 vs. 1.64 ± 0.40 Hz, *p* > 0.05). Meanwhile, 50% (36/72) of LTM neurons were responsive to EA-Ta stimulation, among which the spontaneous firings of 55.6% (20/36) of responsive LTM neurons were markedly increased (post vs. pre: 1.08 ± 0.25 vs. 0.51 ± 0.10 Hz, *p* < 0.01), while 44.4% (16/36) of responsive LTM neurons were slightly depressed, although not statistically significant (post vs. pre: 0.32 ± 0.06 vs. 0.50 ± 0.10 Hz, *p* > 0.05) (Figures 5F–H). Thus, our data suggested that the enhanced spontaneous firing of LTM neurons and inhibited WDR neurons was involved in pain relief in CFA-treated rats.

### EA-Ta Led to a Significant Inverse Correlation in the Discharge Rate of WDR and LTM Neurons

**FIGURE 6:**
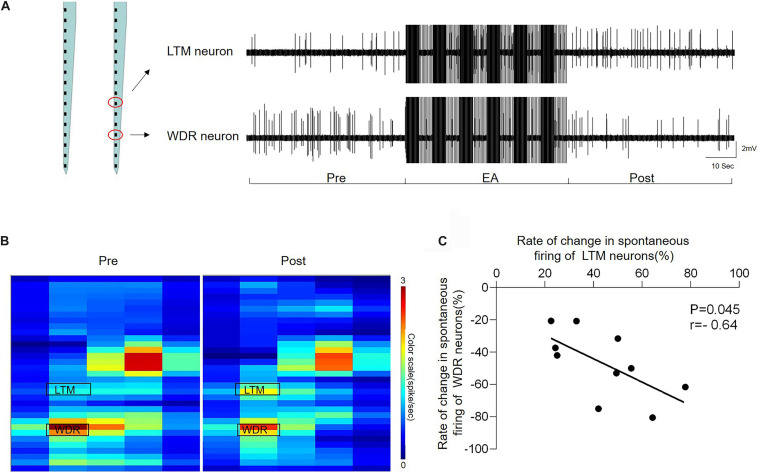
Electroacupuncture-threshold of A fibers led to a significant inverse correlation of the changes in the frequency between LTM and WDR neurons. The heat map and representative traces showed synchronous changes of WDR and LTM neurons before and after EA-Ta. The frequencies of spontaneous firing of WDR and LTM neurons were elicited obviously before EA in CFA rats. EA-Ta applied to ST34 considerably facilitated the spontaneous firing of LTM neurons, while the WDR neurons were suppressed compared with the baseline (Pre-EA) **(A,B)**. The scatter plots displayed the correlation of the discharge rate in spontaneous firing between WDR and LTM neurons **(C)**. Each scatter represented the changes in the frequency for a pair of excited LTM and suppressed WDR neurons recorded synchronously. Pearson’s correlation coefficient of the changes in the frequency between WDR and LTM neurons was statistically significant (*r* = −0.64, *p* = 0.045) **(C)**.

The heat map and representative traces showed synchronous changes in the WDR and LTM neurons (Figures 6A,B). In order to clarify the interaction between the enhanced LTM neurons and inhibited WDR neurons we observed above, we further performed the coherence analysis to determine the correlation of changes in the frequency of inhibited WDR neurons and excited LTM neurons recorded in the same microelectrode array following EA-Ta stimulation. By evaluating the changes in the frequency of synchronous spontaneous firing of WDR and LTM neurons, an inverse correlation between the changes in the frequency of spontaneous firing was observed in WDR and LTM neurons. Pearson’**s** correlation coefficient was statistically significant (*r* = −0.64, *p* = 0.045) (Figure 6C), indicating that the spontaneous firing of WDR neurons was suppressed along with the excitation of LTM neurons following EA-Ta application. These results implied that EA-Ta was likely to alleviate muscle pain, which was associated with facilitation of the spontaneous firing of LTM neurons and inhibition of WDR neuronal activities in the spinal dorsal horn of CFA-treated rats.

## Discussion

In the present study, we showed that inflammatory muscle pain was induced by injection of CFA, which was indicated by imbalance of weight-bearing and abnormal spontaneous EMG activities of the inflamed muscle. Intriguingly, EA-Ta inhibited the abnormal EMG activities and the imbalance of weight-bearing, but EA-Tc exacerbated the spontaneous EMG activities, suggesting that activating peripheral A-fibers was essential for EA analgesia, while activating C-fibers amplified local pain signaling. In accordance with the behavioral phenotypes, the spontaneous firing of LTM neurons was facilitated, while WDR neurons were suppressed by EA-Ta. Notably, coherence analysis showed that the inhibited WDR neurons are correlated with the enhanced LTM neurons. To summarize, these results provided a strong evidence that EA could alleviate inflammatory muscle pain, which was associated with facilitation of the spontaneous firing of LTM neurons and inhibition of WDR neuronal activities in the spinal dorsal horn of CFA-treated rats.

Acupuncture stimulation has been reported to excite various afferent fibers, which are composed of thick myelinated Aα- and Aβ-fibers, thin myelinated Aδ-fibers, and unmyelinated C-fibers. In fact, many human and animal studies have shown that EA with different intensities activated different types of peripheral afferent fibers and produce different extents of analgesia (Xu et al., 2003; Zhu et al., 2004; Xin et al., 2016). Some studies have shown that EA with high-intensity stimulation is more effective than low-intensity stimulation of EA (Barlas et al., 2006; Yu et al., 2018; Lv Z. T. et al., 2019). Conversely, some contrary conclusions have also been reported in animal studies (Lee et al., 2005; Ceccherelli et al., 2008), which implies the necessity to explore the appropriate intensity of EA in the treatment of diseases. Previous studies have demonstrated that acupuncture-induced analgesia resulted from the activation of A-fiber and C-fiber inputs in normal animals, which are mediated by segmental and systemic modulating mechanisms, respectively (Zhu et al., 2004). According to the previous study, the EA stimulation intensity with the threshold for the activation of A-fiber reflex and the threshold for the activation of C-fiber reflex was 1.68 ± 0.53 and 4.78 ± 0.45 mA, respectively. Besides, EA with low-intensity current (1 mA) has been demonstrated to mainly excite A-fibers selectively. In contrast, EA with high-intensity current (4.5 mA) excited C-fibers (Liu et al., 2011). However, the appropriate intensity of EA at local acupoints in CFA rats remained unknown. In this study, the high intensity and low intensity of EA were distinguished by calculating the threshold of activating peripheral A-fibers (Ta) and C-fibers (Tc). We first confirmed that EA with Ta intensity appears to be more effective in inhibiting the abnormal activities of EMG and relieving inflammatory muscle pain. The analgesic effect produced by EA-Ta could be explained by the gate control theory, which was proposed by Melzack and Wall (1965). This theory provided a theoretical framework for explaining pain relief by enhancing inputs of A-fibers within the segment to excite SG cells and elicit inhibition of sensory inputs. Recent studies on peripheral analgesia have shown that electrical stimulation with Ta intensity restored the enhanced nociceptive afferents in models of demyelination and axotomy (Zhu et al., 2012; Chen et al., 2019). These findings suggested that EA could alleviate inflammatory muscle pain *via* activating the A-fiber afferent to close the “gate” and inhibit spinal nociceptive transmission. Additionally, our data demonstrated that nociceptive somatosensory inputs could be suppressed by acupuncture applied to local acupoint, in which the spinal dorsal horn plays a crucial role in proceeding and integrating the inhibitory outcomes (Rong et al., 2005; Lv P. R. et al., 2019).

Dorsal horn neurons receive sensory information from primary afferents and transmit to projection neurons for relay to several brain areas (Todd, 2010). The WDR neurons represent an important component in the network of spinal pain transmission and modulation. These neurons receive a convergence of inputs from the skin, viscera, and muscle (Le Bars, 2002). It was demonstrated that noxious and non-noxious stimuli elicited a progressive increase of WDR neurons response along with the increasing of stimulation intensity. The present study demonstrated that the analgesic effect of EA-Ta may be involved in the inhibition of WDR neurons in the spinal dorsal horn, which is consistent with previous reports (Yu et al., 2019; Wang et al., 2020; Xue et al., 2020).

The neuronal components of the spinal dorsal horn are interconnected by complex synaptic circuits (West et al., 2015; Polgár et al., 2020). In particular, the interlaminar communication is crucial in the processing of nociceptive information (Petitjean et al., 2012; Seibt and Schlichter, 2015). Thus, the discharge activity of a single neuron is insufficient to represent the overall response. Traditional extracellular recordings were performed with a single electrode, which is largely restricted to the observation of individual neuronal properties. In the present study, we recorded neuronal activities in different layers of the spinal dorsal horn simultaneously *via* microelectrode arrays with 32 channels, which are sensitive enough to detect lamina- and region-specific encoding of innocuous and noxious mechanical stimuli. With this method, the density of recording channels was increased without inducing more tissue damage and the spatial resolution is higher than that of the single electrode (Pancrazio and Cogan, 2019). More importantly, application of microelectrode arrays in the spinal cord allows for recording of several locations simultaneously and comparing neurons in different regions of the spinal dorsal horn.

Generally, neurons located in laminae III–IV of the dorsal horn receive inputs mainly from myelinated afferents, while the majority of unmyelinated C-fibers transport nociceptive information to laminae I–II and lamina V of the dorsal horn. Evidence shows that the processing of nociceptive information in the dorsal horn involves interlaminar synaptic interactions in different laminae (Bráz and Basbaum, 2009). Our study demonstrated that the firing rate of LTM neurons in intermediate laminae was correlated with the WDR neurons, which indicated that WDR neurons may be inhibited by interneurons that are excited by non-noxious inputs in the same segment. Accordingly, EA-Ta could activate the A-fiber afferent to alleviate inflammatory muscle pain, which might be associated with activation of the firing of LTM neurons to close the “gate” and inhibit WDR neuronal activities.

Although there are direct synaptic connections in some primary afferents and projection cells, interneurons are the mainly postsynaptic targets for primary afferents (Ganley et al., 2015). Interneurons are involved in modulating modality-specific circuits to ultimately affect our perception of afferent inputs (Yaksh, 1989; Graham and Hughes, 2020). The firing activities of WDR and LTM neurons are modulated by interneurons, which can be divided into two main classes: excitatory and inhibitory. Normally, inhibitory interneurons continuously release GABA to decrease the excitability of neurons and modulate pain transmission. However, the inhibitory neurotransmission can be lost after nerve injury or inflammation (Moore et al., 2002; Hughes and Todd, 2020). In future studies, we will focus on identifying populations of interneurons in different laminae to verify their roles in innervating WDR and LTM neurons.

In summary, the present study demonstrated that EA-Ta was an appropriate intervention to relieve inflammatory muscle pain, which was associated with activation of the firing of LTM neurons and inhibition of WDR neuronal activities in the spinal dorsal horn.

## Data Availability Statement

The original contributions presented in the study are included in the article/supplementary material, further inquiries can be directed to the corresponding authors.

## Ethics Statement

The animal study was reviewed and approved by The Institutional Animal Welfare and Use Committee of the institute of Acupuncture and Moxibustion, China Academy of Chinese Medical Sciences.

## Author Contributions

X-HJ and X-YW designed this experiments. C-LD-M mainly completed this experiments. X-NZ, H-YW, YW, Z-YQ, and HS assisted in the completion of animal behavior experiments. WH and Y-SS provided help in writing and modifying this manuscript. All authors contributed to the article and approved the submitted version.

## Conflict of Interest

The authors declare that the research was conducted in the absence of any commercial or financial relationships that could be construed as a potential conflict of interest.
